# Supplementary material for: Modeling decision-making under uncertainty with qualitative outcomes
Source: PLoS Comput Biol. 2025 Mar 3;21(3):e1012440. doi: 10.1371/journal.pcbi.1012440 (PMC11918403; doi:10.1371/journal.pcbi.1012440)
Supplement: S1 Text — (DOCX) [file pcbi.1012440.s001.docx]

**S1 Text. Hypothetical Medical Scenario**

“You were involved in a car accident. As a result, you suffered from severe spinal injury and lost the motor function of your legs. You were immediately rushed into the hospital and were informed by the doctor that without treatment, your legs would end up completely paralyzed, so that you would be unable to move them at all.

In each trial, you will have to choose one of two suggested treatments that focus on the improvement of motor function of your legs. One of the treatments is conservative and has a certain outcome. The other treatment is experimental and has two possible outcomes, with different probabilities for the occurrence of each one.

For the purpose of this study, the scenario only involves injuries which caused motor function loss. Other injuries (like bruises) you got in this car accident were mild and would recover with simple treatment. So when you make decisions in the "Medical Block", you only have to consider motor function loss of your legs.”
