# Supplementary material for: Modeling decision-making under uncertainty with qualitative outcomes
Source: PLoS Comput Biol. 2025 Mar 3;21(3):e1012440. doi: 10.1371/journal.pcbi.1012440 (PMC11918403; doi:10.1371/journal.pcbi.1012440)
Supplement: S2 Text — (DOCX) [file pcbi.1012440.s002.docx]

**S2 Text. Sensitivity Analysis priors**

Our priors were chosen based on Levy et al. [1] and were set at slight risk aversion (α = 0.7) and moderate ambiguity aversion (β = 0.65). However, we allowed considerable flexibility by setting a relatively large standard deviation. We conducted a sensitivity analysis to compare model fit against models with less informed and uninformed hyper-priors. For the less informed hyper-priors, for risk attitude, we used a scaled beta distribution (0-2) with a mean of 1. For ambiguity attitude, in both the utility and Estimated Value models, we used a normal distribution with a mean of 0, assuming risk and ambiguity neutrality. For the uninformed hyper-priors, we used uniform distributions for both risk and ambiguity attitudes. See S1 Table for results.

1. Levy I, Snell J, Nelson AJ, Rustichini A, Glimcher PW. Neural representation of subjective value under risk and ambiguity. J Neurophysiol. 2010;103: 1036–1047.
