# Supplementary material for: Modeling decision-making under uncertainty with qualitative outcomes
Source: PLoS Comput Biol. 2025 Mar 3;21(3):e1012440. doi: 10.1371/journal.pcbi.1012440 (PMC11918403; doi:10.1371/journal.pcbi.1012440)
Supplement: S1 Table — (DOCX) [file pcbi.1012440.s003.docx]

**S1 Table. Model comparison sensitivity analysis priors**

As shown below, our analysis indicates that the informed hyper-priors provide a slight advantage over the less informed and uniform priors. The difference between the informed and less informed models is minor, likely due to the robustness of our model to these changes. The more substantial difference between the informed and uninformed models highlights the importance of incorporating prior knowledge in shaping our analysis.

## <https://github.com/KoremNSN/QualMod/blob/main/4.1.PriorSensCheck.ipynb>

|  | Rank | LOO | p_loo | d_loo | Weight | SE |
| --- | --- | --- | --- | --- | --- | --- |
| Monetary, Utility |  |  |  |  |  |  |
| Informed | 0 | -1910.83 | 142.76 | 0 | 0.50 | 42.37 |
| Less Informed | 1 | -1911.94 | 147.29 | 1.11 | 0.50 | 42.69 |
| Uninformed | 2 | -2063.47 | 124.79 | 152.65 | 0 | 42.02 |
| Monetary, Estimated Value |  |  |  |  |  |  |
| Informed | 0 | -1563.26 | 225.65 | 0 | 0.50 | 45.63 |
| Less Informed | 1 | -1563.94 | 221.14 | 0.68 | 0.50 | 45.12 |
| Medical, Estimated Value |  |  |  |  |  |  |
| Informed | 0 | -1411.89 | 210.83 | 0 | 0.50 | 45.14 |
| Less Informed | 1 | -1415.10 | 210.79 | 3.21 | 0.50 | 44.86 |
