# Supplementary material for: Modeling decision-making under uncertainty with qualitative outcomes
Source: PLoS Comput Biol. 2025 Mar 3;21(3):e1012440. doi: 10.1371/journal.pcbi.1012440 (PMC11918403; doi:10.1371/journal.pcbi.1012440)
Supplement: S3 Text — (DOCX) [file pcbi.1012440.s004.docx]

**S3 Text. Sensitivity Analysis Categorical**

While the outcomes in our study were categorical, they were ordinal in nature, as “moderate improvement” is objectively better than “slight improvement.” Accordingly, our model assumes that each successive category holds the same or a higher value. To capture this structure, we used a truncated normal distribution with a mean of 4, a standard deviation (SD) of 2, and a lower limit of 0; we refer to this as the “ordinal model.” We introduced two alternative models to assess whether this assumption holds and demonstrate the model’s ability to handle “pure” categorical data. The first, the “semi-categorical model,” follows the same additive approach but uses a normal distribution without a lower bound, allowing categories to take on negative values. The second, the “pure categorical model,” assigns a distinct value to each category (see S5 Equation). However, this approach is not ideal for our data, as the incremental nature of the outcomes makes the relationship between the mean and SD critical—when the mean increases, so does the SD. See S1 Equation and S2 Table for detailed results.
