# Supplementary material for: Modeling decision-making under uncertainty with qualitative outcomes
Source: PLoS Comput Biol. 2025 Mar 3;21(3):e1012440. doi: 10.1371/journal.pcbi.1012440 (PMC11918403; doi:10.1371/journal.pcbi.1012440)
Supplement: S1 Equation — (DOCX) [file pcbi.1012440.s005.docx]

**S1 Equation. Full categorical model**

$$SV=(P-ꞵ*\frac{A}{2})*v_{i}$$

$$\beta\sim Normal (\mu_{\beta}, \sigma_{\beta},-1.5, 1.5)$$

$$\nu_{i}\sim Normal (\mu_{\nu_{i}},2,0,\infty)$$

$$\mu_{\beta}\sim Normal (0.65,1)$$

$$\sigma_{\beta}\sim Gamma (2, 1)$$

$$\mu_{\nu_{1}}\sim Normal (5, 2,\infty, \infty)$$

$$\mu_{\nu_{1}}\sim Normal (12, 2,\infty, \infty)$$

$$\mu_{\nu_{1}}\sim Normal (20, 2,\infty, \infty)$$

$$\mu_{\nu_{1}}\sim Normal (25, 2,\infty, \infty)$$
