# Supplementary material for: Modeling decision-making under uncertainty with qualitative outcomes
Source: PLoS Comput Biol. 2025 Mar 3;21(3):e1012440. doi: 10.1371/journal.pcbi.1012440 (PMC11918403; doi:10.1371/journal.pcbi.1012440)
Supplement: S2 Table — (DOCX) [file pcbi.1012440.s006.docx]

**S2 Table. Model comparison sensitivity analysis categorical**

## Based on the LOO and p_loo values, the ordinal model best fits the data across both tasks and datasets. Although the difference in LOO between the ordinal and semi-categorical models is relatively small, the higher complexity of the semi-categorical model is reflected in its p_loo, resulting in the ordinal model receiving higher model weights. This indicates that the ordinal model better balances fit and complexity.

## The pure categorical model shows the poorest fit, which aligns with the nature of our dataset, where the outcomes follow an ordinal structure. Additionally, the standard deviations (SD) were set to 2 in the pure categorical model to improve convergence and matrix stability. Overall, the results confirm that the ordinal model presented in the main article provides the best fit and demonstrates that this framework can also be extended to test pure categorical data when needed.

For the complete analysis and comparison of the estimated values, see https://github.com/KoremNSN/QualMod/blob/main/4.2.OrdinalSensCheck.ipynb

|  | Rank | LOO | p_loo | d_loo | Weight | SE |
| --- | --- | --- | --- | --- | --- | --- |
| In-person Sample |  |  |  |  |  |  |
| Monetary |  |  |  |  |  |  |
| Ordinal | 0 | -1560.88 | 222.99 | 0 | 0.92 | -1560.88 |
| Semi-Categorical | 1 | -1567.79 | 240.24 | 6.91 | 0.08 | -1567.79 |
| Pure Categorical | 2 | -1610.42 | 203.67 | 49.53 | 0 | -1610.42 |
| Medical |  |  |  |  |  |  |
| Ordinal | 0 | -1411.57 | 210.6 | 0 | 0.75 | -1411.57 |
| Semi-Categorical | 1 | -1416.03 | 226.22 | 4.46 | 0.25 | -1416.03 |
| Pure Categorical | 2 | -1439.44 | 186.01 | 27.87 | 0 | -1439.44 |
| Online Sample |  |  |  |  |  |  |
| Monetary |  |  |  |  |  |  |
| Ordinal | 0 | -3790.65 | 825.45 | 0 | 0.96 | -3790.65 |
| Semi-Categorical | 1 | -3818.56 | 898.68 | 27.91 | 0.04 | -3818.56 |
| Pure Categorical | 2 | -3950.99 | 777.15 | 160.34 | 0 | -3950.99 |
| Medical |  |  |  |  |  |  |
| Ordinal | 0 | -4103.18 | 788.57 | 0 | 0.52 | -4103.18 |
| Semi-Categorical | 1 | -4104.29 | 868.76 | 1.11 | 0.48 | -4104.29 |
| Pure Categorical | 2 | -4302.66 | 722.04 | 199.48 | 0 | -4302.66 |
