# Supplementary material for: Modeling decision-making under uncertainty with qualitative outcomes
Source: PLoS Comput Biol. 2025 Mar 3;21(3):e1012440. doi: 10.1371/journal.pcbi.1012440 (PMC11918403; doi:10.1371/journal.pcbi.1012440)
Supplement: S4 Text — (DOCX) [file pcbi.1012440.s007.docx]

## **S4 Text. Surgery Analysis**

## Previous experience can influence ambiguity attitudes. Therefore, in the in-person dataset, we asked participants whether they had a history of major surgeries. We ran the model separately for each group to assess the impact of past surgical experience on ambiguity attitudes in the medical domain and cross-domain associations. We then compared the posterior distributions of the ambiguity attitude parameter (β) between groups and examined the posterior distribution of the association between monetary and medical ambiguity attitudes.

We found no robust difference in the ambiguity attitude parameter (β) between participants with and without a history of surgery (mean difference: -0.062, 89% HDP [-0.15, 0.03]; S1 Figure). However, the data suggests the need for further investigation (e.g., increasing the sample size).

When examining the association between ambiguity attitudes in the monetary and medical domains, we found robust associations in both the Surgery (mean slope = 1.086, 89% HDP [0.779, 1.385] S2A Figure) and No surgery (mean slope = 0.586, 89% HDP [0.462, 0.715] S2B Figure) groups.
