# Supplementary figures and images for: Modeling decision-making under uncertainty with qualitative outcomes
Source: PLoS Comput Biol. 2025 Mar 3;21(3):e1012440. doi: 10.1371/journal.pcbi.1012440 (PMC11918403; doi:10.1371/journal.pcbi.1012440)

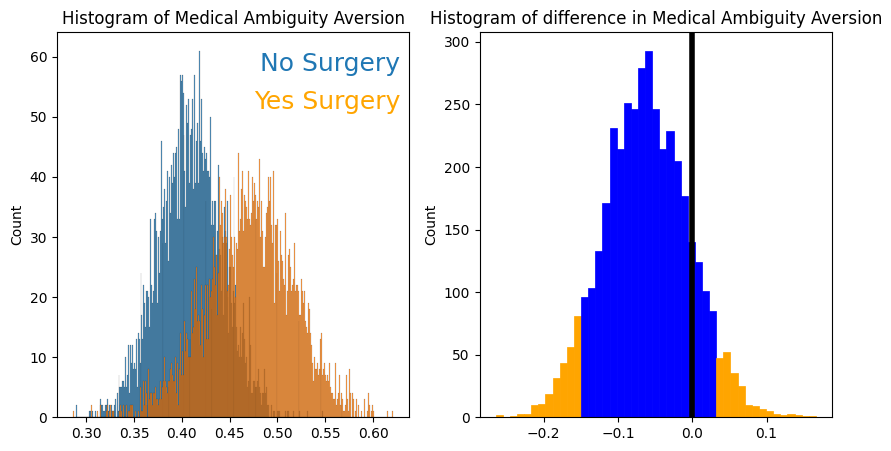

Supplement: S1 Fig — (TIF) [file pcbi.1012440.s008.tif]

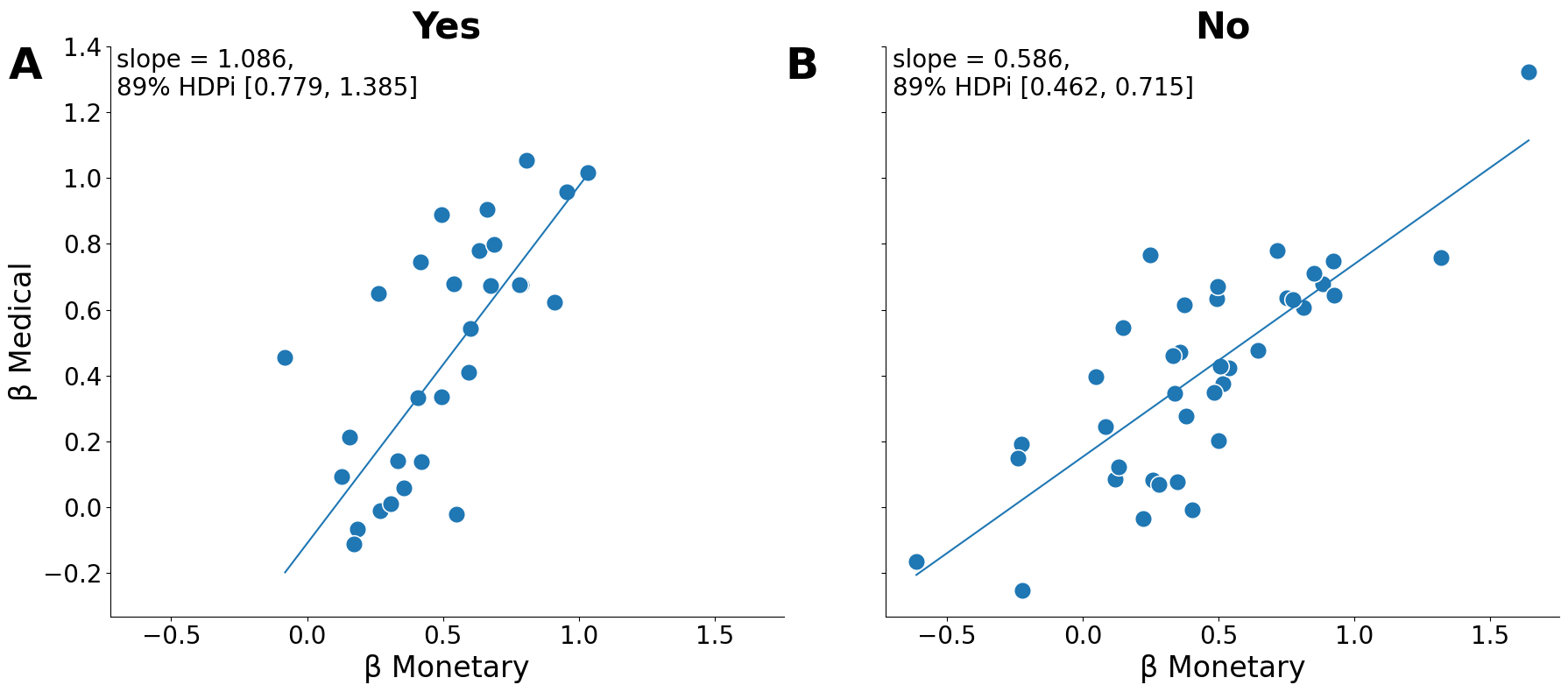

Supplement: S2 Fig — (TIF) [file pcbi.1012440.s009.tif]

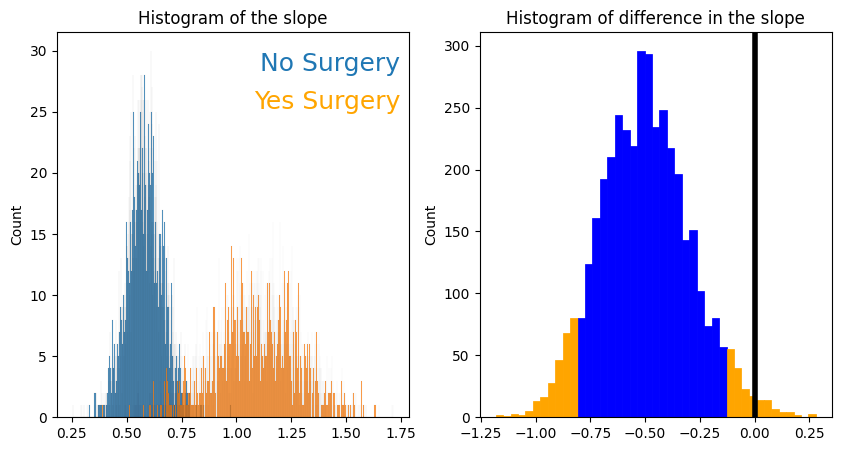

Supplement: S3 Fig — (TIF) [file pcbi.1012440.s010.tif]

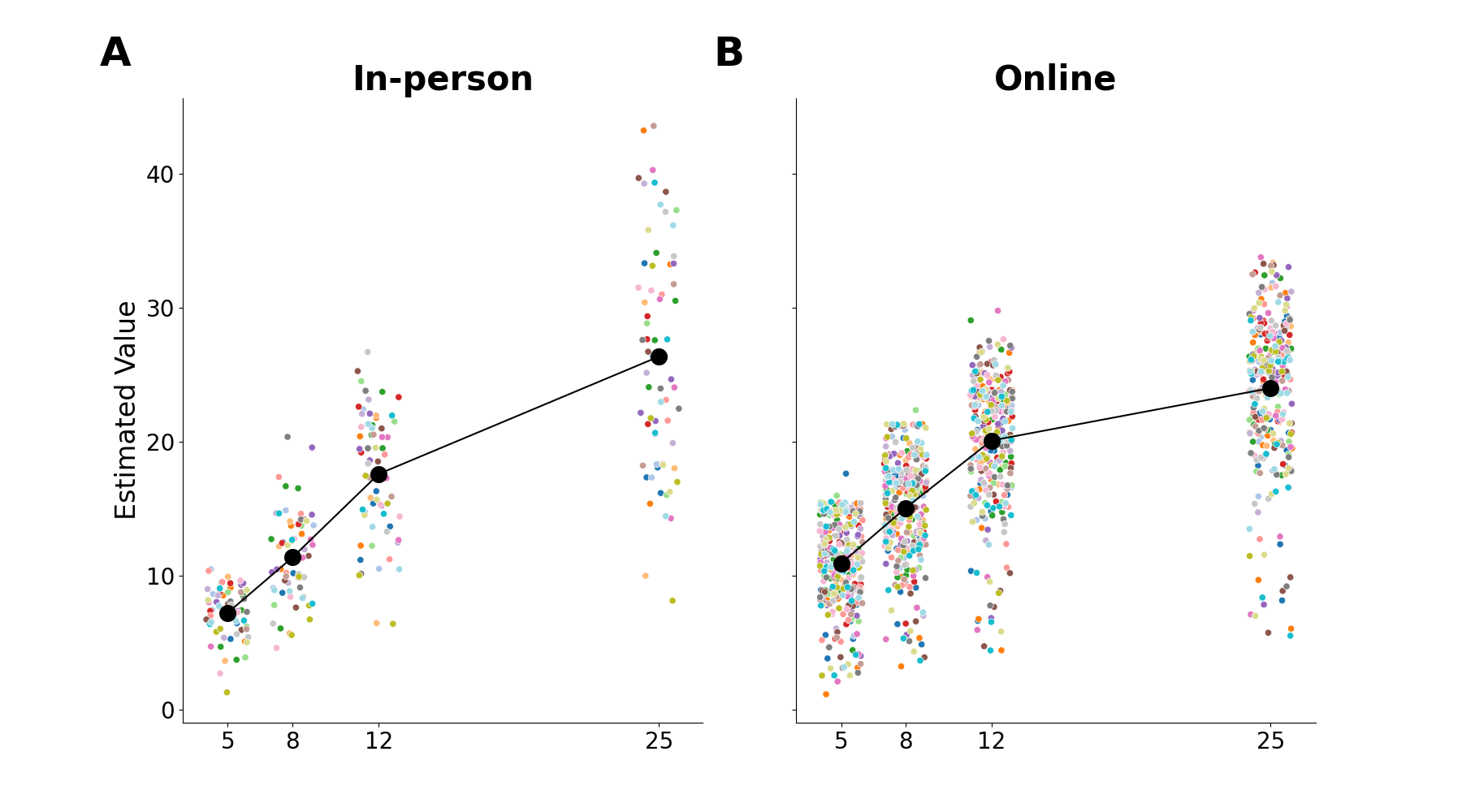

Supplement: S4 Fig — (TIF) [file pcbi.1012440.s011.tif]
