# Supplementary material for: Modeling decision-making under uncertainty with qualitative outcomes
Source: PLoS Comput Biol. 2025 Mar 3;21(3):e1012440. doi: 10.1371/journal.pcbi.1012440 (PMC11918403; doi:10.1371/journal.pcbi.1012440)
Supplement: S5 Text — (DOCX) [file pcbi.1012440.s012.docx]

## **S5 Text. Simulation Analysis**

As models become more complex, their ability to fit the data might result from overfitting. Overfitting occurs when a model learns the noise and details in the training data to the extent that it performs well on the training data but poorly on new, unseen data. Thus, overfitting results in a model that cannot be generalized. Several methods can reduce the likelihood of overfitting. One approach is to replicate the results using an independent dataset, such as the online sample used in the main paper. Another approach is through simulations.

In this analysis, we simulated choices based on the utility function (see Equation 1 in the main article) with different noise levels and numbers of participants. Specifically, we used sample sizes (N) of 30, 60, 120, and 300, and noise levels of 0.1, 0.3, and 0.5. See S3 Table for results.
