# Supplementary material for: Modeling decision-making under uncertainty with qualitative outcomes
Source: PLoS Comput Biol. 2025 Mar 3;21(3):e1012440. doi: 10.1371/journal.pcbi.1012440 (PMC11918403; doi:10.1371/journal.pcbi.1012440)
Supplement: S3 Table — (DOCX) [file pcbi.1012440.s013.docx]

**S3 Table. Model Comparisons Simulation Analysis**

As shown below, our analysis indicates that with increased noise, the Estimated Values model tends to perform better compared to the Classic Utility model. This suggests that the Estimated Values model is more robust to noise regardless of sample size or when the choices are generated using a different model.

Code: https://github.com/KoremNSN/QualMod/blob/main/5.simulation_model_check.ipynb

|  | Rank | LOO | p_loo | d_loo | Weight | SE |
| --- | --- | --- | --- | --- | --- | --- |
| N = 30, noise = 0.1 |  |  |  |  |  |  |
| Classic Utility | 0 | -1112.09 | 61.46 | 0 | 0.80 | 26.66 |
| Estimated Value | 1 | -1126.33 | 79.41 | 14.24 | 0.20 | 27.40 |
| N = 30, noise = 0.3 |  |  |  |  |  |  |
| Classic Utility | 0 | -1312.98 | 74.11 | 0 | 0.68 | 28.15 |
| Estimated Value | 1 | -1322.97 | 86.14 | 9.99 | 0.32 | 26.50 |
| N = 30, noise = 0.5 |  |  |  |  |  |  |
| Estimated Value | 0 | -1465.87 | 88.06 | 0 | 0.57 | 23.66 |
| Classic Utility | 1 | -1471.86 | 74.33 | 5.99 | 0.43 | 25.74 |
| N = 60, noise = 0.3 |  |  |  |  |  |  |
| Classic Utility | 0 | -2740.98 | 127.66 | 0 | 0.66 | 36.68 |
| Estimated Value | 1 | -2754.77 | 158.89 | 13.79 | 0.34 | 34.27 |
| N = 60, noise = 0.5 |  |  |  |  |  |  |
| Estimated Value | 0 | -2855.99 | 177.70 | 0 | 0.73 | 35.26 |
| Classic Utility | 1 | -2889.43 | 147.48 | 33.44 | 0.27 | 37.14 |
| N = 120, noise = 0.5 |  |  |  |  |  |  |
| Estimated Value | 0 | -5758.29 | 342.84 | 0 | 0.64 | 46.90 |
| Classic Utility | 1 | -5810.99 | 291.50 | 52.70 | 0.36 | 52.12 |
| N = 300, noise = 0.5 |  |  |  |  |  |  |
| Estimated Value | 0 | -14463.39 | 844.34 | 0 | 0.68 | 74.19 |
| Classic Utility | 1 | -14609.81 | 710.70 | 146.42 | 0.32 | 82.35 |
