# Supplementary material for: Modeling decision-making under uncertainty with qualitative outcomes
Source: PLoS Comput Biol. 2025 Mar 3;21(3):e1012440. doi: 10.1371/journal.pcbi.1012440 (PMC11918403; doi:10.1371/journal.pcbi.1012440)
Supplement: Attachment — Submitted filename: Response to Rev2.docx [file pcbi.1012440.s016.docx]

**Reviewer #1**

1. To re-state a point from the first review – the design of the experiment requires a step that translates the qualitative experience onto a quantitative monotonic linear scale. This appears useful here, and the findings are compelling. But the methods and contributions of the work should then be represented more precisely. The model in the paper still uses (relies on?) “numerical data,” just like the description of traditional models in the authors’ summary and the risk papers cited in the introduction. (In the discussion, sentence 208 qualifies that the paper is about choices between “ordinal outcomes.” These came from the simplified recovery scale, but the subsequent paragraph argues against oversimplified quantifications of qualitative outcomes, which is an odd juxtaposition.) My overall conceptual concern is that the ability to compare qualitative fields is still sometimes described in the paper as a contribution arising specifically from the *modeling* ( e.g., “leveraging computational modeling to extract values from qualitative outcomes”) rather than also the gathering of quantified data in the experiment and/or overall research. This needs to be described more carefully to understand future applications and generalizations.
   a. Response: Thank you for the thoughtful feedback. We understand the potential confusion and have clarified this in the revised manuscript. Specifically, this study focuses on estimating the value of ordinal **qualitative** **outcomes** while using **quantitative probabilities** (e.g., 75%) rather than qualitative descriptions of risk (e.g., “a high chance to win”). This distinction has been emphasized throughout, and we revised the discussion to clarify any misconception. While our study does not address qualitative probabilities, the key innovation lies in handling ordinal outcomes flexibly without oversimplifying them into fixed quantities.

We also refined the framing to highlight that the contributions stem from the experimental design (which collects ordinal data) and the modeling approach (which quantifies it). The Bayesian framework preserves uncertainty through distributions, avoiding oversimplifications inherent in traditional methods. Additionally, we softened claims about broader applications to qualitative risk/ambiguity, as these remain beyond the scope of this study.

As noted in the previous revision, supplementary analyses include models treating outcomes as categorical and independent. However, the main model remains grounded in ordinal data, which best fits the study's structure. We hope this provides greater clarity regarding the contributions and limitations of our work.

b. In the text:
   i. “When presented quantitatively, uncertainty around choice outcomes can be categorized into two components: risk and ambiguity.” Lines: 39-40
   ii. “Importantly, uncertainty in both domains was presented quantitatively (e.g., a 50% chance) rather than qualitatively (e.g., high chance). This allowed us to examine the subjective valuation of qualitative outcomes independently from qualitative risk representations.” Lines: 66-67
   iii. “In the medical domain, fitting the utility function is challenging due to the qualitative nature of the outcomes, which lacked a quantifiable value for the value (V) component of the model (although uncertainty was still presented quantitatively).” Lines: 120-122
   iv. “Theoretically, this problem stems from the difficulty in comparing outcomes with unknown cardinal values [30].” Lines: 221-222

2. Perhaps a facet of the above issue, although there the paper uses ordinal outcome data, the illustrative example of what the model could do in the introduction is nominal (Thai vs. Italian). It’s less clear to me that this broader claim is sufficiently demonstrated in the present work. Indeed, the response letter describes nominal (apples/oranges) data as a future application. The absence of a need for fixed distances in the measurements certainly speaks to the feasibility of categorical/nominal applications, as do the categorical models in the supplement, but there is not *evidence* demonstrated in the paper for it. If the broader claim about all qualitative data is made, it seems reasonable to expect findings that speak to it. To be clear, I find the paper compelling. I also feel it is critical to be precise in terms of what this specific work has proven/shown versus what it shows capability/ feasibility/ promise to do.
   a. Response: Thank you for the insightful feedback. We agree with the reviewer and have adjusted the text to be more precise about the scope of the current study. Specifically, we now emphasize that this work demonstrates the ability to model **ordinal qualitative outcomes** and does not provide direct evidence for applications to **nominal data** (e.g., Thai vs. Italian). While the absence of fixed distances in our framework supports the feasibility of applying it to categorical/nominal data, this remains a potential future application rather than a demonstrated result in the present work.

To address this, we have revised the introduction and other sections to align more closely with the current model and data, reducing the emphasis on broader claims about all qualitative data. We hope this provides a clearer delineation between the demonstrated findings and the framework's potential.

b. In the text:
   i. “Many decisions, however, are qualitative, such as choosing between your regular coffee or paying more for premium coffee beans.” Lines: 34-35
   ii. “By estimating these values, we can examine individual differences and use them in more precise parametric analyses, such as with neural representation of value, enhancing our understanding of the neural mechanisms involved in processing uncertainty.” Lines: 231-234

3. From the values reported in the results section, it seems that the recovery data also shows diminishing marginal value as the scale increases (assuming linearity of the underlying scale), similar to the monetary data. Is that correct? Note the question in the prior review about how participants might *interpret* the scale remains regardless of whether an expert-designed it to be linear. Would it be useful to comment on that parallel subjective structure between the monetary and health perceptions?
   a. Response: Thank you for this thoughtful comment. The X-axis for the medical domain is indeed arbitrary, which is why we chose to plot both the monetary and medical domains as categories rather than relying on objective values for the monetary domain. Without an objective underlying scale for the medical outcomes, we cannot make claims about diminishing marginal values or whether there are diminishing returns.
4. I’m a little confused by the statement that cumulative prospect theory is not equipped to handle qualitative outcomes. Is that meant literally in terms of the equations (rather than conceptually/theoretically)? For example, prospect theory (and thus presumably CPT) predicts diminishing marginal returns in subjective value, which seems consistent with what was observed in the qualitative recovery data (see prior question)? I apologize if I’m missing some context to this argument- it seems like integrating these findings with prior theory would be a positive benefit.
   a. Response: Thank you for raising this point. Yes, our statement is meant literally in terms of the equations. While our formula and theoretical framework share similarities with cumulative prospect theory (CPT), our main contribution lies in implementing a tool that accommodates ordinal qualitative outcomes. All code is freely available online, making it easy for researchers to explore and adapt.

We have revised the text to clarify that our approach differs from CPT more in its **practical application** than in the underlying theoretical principles of how value is derived. This distinction ensures that our contribution is positioned as complementary to prior theory while offering a new framework for handling qualitative data.

b. In the text:
   i. “Current models, such as cumulative prospect theory [22], are not practically equipped to handle qualitative outcomes.” lines: 53-54

5. Upon re-review, and with the addition of discussion of products, the authors should probably cite the following paper : Muthukrishnan, A. V., Luc Wathieu, and Alison Jing Xu. "Ambiguity aversion and the preference for established brands." Management Science 55, no. 12 (2009): 1933-1941. As the title implies, the paper shows that ambiguity aversion in financial gambles correlates with ambiguity aversion in brands (e.g. preferring established brands vs. newer ones.)
   a. Response: Thank you for the excellent suggestion. We now include the citation for Muthukrishnan, Wathieu, and Xu (2009) in the revised manuscript, as it aligns well with the discussion on ambiguity aversion and its applications to product preferences. We appreciate the opportunity to integrate this relevant reference into our work.
   b. In the text: “Additionally, ambiguity aversion revealed in monetary decisions can influence preferences in other domains, such as a tendency to favor established brands over newer alternatives [43], particularly when attribute uncertainty is high.” Lines: 271-273
6. The finding that experience with surgery improves cross-domain association seems to be an important theoretical point. (This was more of a comment than a question. But despite not being central to the computational model, might it be valuable to move that analysis to the main paper?)
   a. Response: Thank you for highlighting this promising and interesting result. While we agree that the finding regarding surgery experience and cross-domain associations is theoretically important, we believe moving it to the main paper could shift the focus away from the primary goals of the study. Additionally, this analysis was exploratory and not part of the original plan. Since the replication sample did not include data on medical history, we were unable to replicate this finding. We appreciate your insightful comment and contribution, which has helped refine the focus and robustness of the paper. Your comment has also inspired new research ideas that we are currently pursuing.

**Reviewer #2**

1. The authors have produced a responsive revision, one that retains the considerable strengths of the original version, but the message is more accessible and more impressive. The strengths include applying sophisticated computational modeling to decision-making under risk and ambiguity comparing alternative models. There are noteworthy results, among them that qualitative outcomes can be modeled using techniques also applied to quantitative outcomes and that ambiguity attitudes generalize across very different domains (money and medical outcomes). The choice of medical outcomes is especially important (degrees of recovery due to treatment). Similar results are obtained across independent samples (online and in-person). The methods are rigorous (e.g., counterbalancing quantitative and qualitative decisions, etc.).
   a. Response: We sincerely thank the reviewer for the thoughtful and encouraging feedback. We are grateful for the acknowledgment of the strengths in our work, including the application of sophisticated computational modeling, the generalization of ambiguity attitudes across domains, and the rigorous methods employed. Your recognition of the importance of modeling qualitative outcomes, particularly in the medical context, and the robustness of our findings across independent samples is greatly appreciated. Thank you for your kind words and support.
2. The authors have addressed the issues of explanation as opposed to description. Their responses have enhanced and clarified their message. The Discussion especially is greatly enhanced, and it now flows beautifully and communicates important conclusions. The revised manuscript is well above threshold—except for a couple of accuracy issues that are easily addressed; see below. However, although the modeling fits within the tradition of psychophysical approaches, I hope that the authors continue to think about explanation. The authors make an excellent case that their models describe a wide range of observed phenomena, more than satisfying the technical requirements of modeling. With powerful and flexible models, it is possible to describe a range of behaviors—and this is no mean feat—in terms of these psychophysical parameters, but the extent to which these parameters actually explain (as contrasted with describe) these behaviors remains a question not only for this work but for much contemporary work on decision making. Predicting decoy and other effects is fantastic, but it is predicting known results; ideally, the best theories predict new, counterintuitive effects. In addition, beyond these data, the authors do not account for some findings that dispute these psychophysical models, but that is beyond the scope of the present work. The authors should continue to think about these issues, but they are operating in a venerable tradition nevertheless.
   a. Response: We thank the reviewer for their thoughtful and encouraging feedback. We fully agree that this work builds upon a substantial body of existing knowledge and is part of a broader tradition of psychophysical modeling. While this study focuses on describing and explaining observed phenomena, we share the reviewer’s aspiration for models that predict new and counterintuitive effects. We are already pursuing this direction, and the models developed in this work serve as the foundation for ongoing efforts to advance predictive capabilities. We greatly appreciate the reviewer’s insights and acknowledgment of the significance of this approach. We have added a more direct reference to the limitations of the current approach and the potential direction for future work.
   b. In the text: “Nevertheless, while this model describes the observed data well, future applications should aim to extend its predictive capabilities to describe behavior more comprehensively. In particular, our experimental design relied on objectively ordinal outcomes (for example, moderate improvement is, by definition, better than slight improvement). A similar modeling approach could be used more generally for inferring values of options that are not inherently better or worse than each other (such as apples and oranges), but this should be empirically tested.” Lines: 234-241
3. As for the accuracy issues, first, the use of the word “only” is a major problem in the following sentence: “The fuzzy-trace theory posits that patients often hold only a gist representation of the outcomes they are considering [37].” To be clear, fuzzy-trace theory does not say that only gist is considered. On the contrary, fuzzy-trace theory says that multiple gist representations and a verbatim representation are encoded (and stored and retrieved) in parallel (e.g., see Reyna, 2012; Reyna, Müller, & Edelson, 2023). However, the tendency to lean more on the simplest gist representation accounts for many empirical phenomena. Because the gist representation is ultimately determinative of behavioral phenomena (e.g., the response pattern in gain-loss framing, the Allais effect, etc.) does not mean that it is the only representation that is processed. Metaphorically, gist is often (for theoretically specified reasons; see papers above) the deciding vote but it is not the only vote, and all of the processes in fuzzy-trace theory contribute to behavior, not just the simplest gist. The following would be accurate and can be substituted for the problem sentence with “only”: “The fuzzy-trace theory posits that patients emphasize the gist representation of outcomes they are considering in decision-making, despite also processing the verbatim representation simultaneously [37].”
   a. Response: We thank the reviewer for clearly articulating precisely what we have meant to say, and pointing out this necessary clarification. We apologize for misrepresenting the fuzzy-trace theory in the original text. In the revised manuscript, we have amended our explanation to accurately reflect the theory, highlighting that gist and verbatim representations are processed in parallel, with decision-making often emphasizing the gist representation. We appreciate the reviewer’s detailed feedback and the suggested correction, which has been incorporated into the revised version to ensure accuracy.
   b. In the text: “The fuzzy-trace theory posits that patients emphasize the gist representation of outcomes they are considering in decision-making despite also processing the verbatim representation simultaneously [37–39].” Lines: 253-255
4. The second accuracy correction per p. 30: “Furthermore, the increased cross-domain association in ambiguity attitudes observed in individuals with prior surgical experience supports the transition from gist to verbatim processing [38].” The transition in the cited paper is exactly the opposite, from relying more on verbatim processing (in novices) to relying more on gist processing (in experts). Developmental data as well as data on novice-expert transitions supports the direction from verbatim to gist. The authors’ results discussed here also support this direction from verbatim to gist. So simply reversing the phrases works to correct this problem, as follows, which is probably what the authors meant: “Furthermore, the increased cross-domain association in ambiguity attitudes observed in individuals with prior surgical experience supports the transition from verbatim to gist processing [38].”
   a. Response: We thank the reviewer for identifying this error and providing a clear and accurate correction. We apologize for the oversight. In the revised manuscript, we have amended the text to accurately reflect that the transition is from verbatim to gist processing, aligning with the cited paper and supporting data. We appreciate the reviewer’s support and detailed feedback.
   b. In the text: “Furthermore, the increased cross-domain association in ambiguity attitudes observed in individuals with prior surgical experience supports the transition from verbatim to gist processing [40].” Lines: 259-261
5. However, this then does present a problem for the sentence that follows: “This finding suggests that personal experience may reinforce a shift towards more detailed, quantitative representations, enriching our understanding of how experience shapes the processing of uncertainty in critical health decisions.” The authors should consider whether their results support this sentence (I think not) and it does not agree with other developmental research on experience/expertise. They should edit the sentence in some way, otherwise it is a non sequitur, or simply delete it.
   a. Response: Following further reading and consideration, we agree with the reviewer’s statement. While our findings suggest that experience increases alignment between the two domains, they do not provide evidence about which system—gist or verbatim—ultimately drives decision-making. It is possible, as the reviewer suggested, that experience shifts the emphasis toward gist processing, thereby increasing alignment with monetary decisions. We have amended the sentence in question in the revised manuscript to reflect this nuance.
   b. In the text: “This finding suggests that, similar to experts, personal experience may facilitate a transition toward greater reliance on gist-based processing, enriching our understanding of how experience shapes the processing of uncertainty in critical health decisions.” Lines: 261-264
6. Reviewer 1’s points were insightful and the authors’ responses to them enhanced the manuscript. Per the reviewer’s comment “To emphasize the usefulness of making cross-domain comparisons of ambiguity, it seems helpful to cite evidence for the benefits of such approaches as shown in cross-domain variance in risk (e.g., Blais, A. R., & Weber, E. U. (2006). A Domain-Specific Risk-Taking (DOSPERT) scale for adult populations. Judgment and Decision making, 1(1), 33-47.)” The authors do a good job of bringing in this important paper without confusing readers. To wit, the DOSPERT scale was developed to contrast differences between domains. Empirical work has subsequently shown that there is cross-domain shared variance, as Blaise and Weber acknowledge, but their emphasis and the whole point of their paper was that domains are different and that generalizing across domains is problematic. The authors navigate this potential confusion well.
   a. Response: We agree that this was an excellent comment, and we made a concerted effort to incorporate this critical paper into our theoretical framework. We appreciate the recognition that we successfully navigated the potential confusion around cross-domain comparisons, emphasizing both the shared variance and the challenges of generalizing across domains, as highlighted by Blais and Weber.
7. In the future, although modeling metrics often combine goodness-of-fit with penalties for the number of parameters, the authors should separately consider how well each model fits the data and then whether added parameters are merited by the current data or external data. The overall metrics are mechanical, whereas a good theorist distinguishes less important and more important parameters and whether parameters (or assumptions) are needed to understand a phenomenon. No revisions are needed here to address this issue but it is something to keep in mind for future work. In sum, this is an impressive paper with relevance to fundamental issues of measurement and theory in decision-making.
   a. Response: We sincerely thank the reviewer for this meaningful feedback and their thoughtful insights, which have significantly enhanced the accessibility, theoretical grounding, and clarity of the paper’s implications. We appreciate the suggestion to evaluate goodness-of-fit and parameter relevance separately in future work. We will keep this in mind as we refine and expand our modeling approaches.
