# Supplementary material for: Modeling decision-making under uncertainty with qualitative outcomes
Source: PLoS Comput Biol. 2025 Mar 3;21(3):e1012440. doi: 10.1371/journal.pcbi.1012440 (PMC11918403; doi:10.1371/journal.pcbi.1012440)
Supplement: Attachment — Submitted filename: Response to reviewers.docx [file pcbi.1012440.s015.docx]

Response to Reviewers

# Reviewer #1:

1. Intro (and discussion): It would be helpful to be more precise in the description of what is considered qualitative. **First**, the uncertainty is still expressed numerically and therefore quantitatively. **Second**, while the description of the medical outcomes are qualitative, the outcome variable still has an exogenously determined quantitative “objective” ordering, so sidesteps the challenge of finding a common metric mentioned on p25. Specifically, the stimuli use a quantitative scale, imposing a translation to a quantitative stepped measure for the participant (which allows for the model in Eq 5, and facilitates extracting subjective values). This can be contrasted with studies on food preferences which may not have a prior orderings. Those studies are often designed to first measure individually defined subjective preferences for foods and then convert those ratings into a quantitative estimate/common currency with more flexibility. It also allows for indifference. This work could also be contrasted with choices between two medications that led to different types of side effects, and the question of whether it’s similarly possible to estimate subjective values in that case.
   a. **Response:** This is a great point. Indeed, our study looked at qualitative outcomes, with the outcome probability and ambiguity still quantitative. We now clarify this more explicitly in the text. Moreover, our qualitative outcomes are ordered. Based on our ordinal scale, we used a truncated normal distribution with a limit of 0 - which can only model positive or indifference. We now added in the supplementary materials, a comparison with a model with no constraints, that is, the value can be negative, and there is no predefined ordering of the outcomes. Comparison between these models results in the ordinal model showing a better fit for both in-person and online samples in both monetary and medical tasks, probably primarily due to lower complexity. As the model compares categories, using the non-ordinal model (value is estimated using a normal instead of truncated normal) will allow us to compare food items' values (e.g., would you rather have an apple or 50% for two oranges). Given enough data, using this model, we can estimate the value of each outcome (e.g., apples, oranges) for each individual. This would be a tremendous future application of our model, which we now mention in the discussion.
   b. In the text:
      i. “Note, however, that the uncertainty levels associated with each outcome were presented quantitatively.” lines 75-76
      ii. “To align with the monetary task, these outcomes were created on an ordinal scale.” lines 400-401
      iii. “Given the ordinal nature of the outcomes, the model employs a truncated normal distribution for each value. See supplementary analysis for a comparison with categorical models.” lines 120-121
      iv. “uncertainty attitudes when making choices between ordinal outcomes.” lines 207-208
      v. “In contrast, our model allows for the introduction of complete qualitative outcomes. Nevertheless, our model still uses quantitative probabilities; in future research, it will be interesting also to include qualitative information about outcome likelihood (for example, “a high chance for success”).” Lines 235-238
      vi. “Using the categorical variant, the model can compare discrete outcomes (e.g., comparing apples to oranges, choosing between an apple, or a 50% chance of two oranges).” lines 331-333
2. Literature: To emphasize the usefulness of making cross-domain comparisons of ambiguity, it seems helpful to cite evidence for the benefits of such approaches as shown in cross-domain variance in risk (e.g., Blais, A. R., & Weber, E. U. (2006). A Domain-Specific Risk-Taking (DOSPERT) scale for adult populations. Judgment and Decision making, 1(1), 33-47.) It may also be helpful to mention research comparing novel (e.g. uncertain) to known (e.g. certain) products and/or brands as examples of research on qualitative ambiguity, since they could be considered analogous to the certainty-equivalent paradigm in the present work.
   a. Response: This is a great suggestion. In the revised manuscript, we talk about these topics.
   b. In the text:
      i. “While self-report questionnaires examined risk-taking across various domains [17], only a few studies quantified individual attitudes in non-monetary domains. Importantly, these studies still employed quantitative outcomes”. Lines 45-48
      ii. “Beyond healthcare, our model can be applied in areas such as marketing, where product comparisons—particularly for new products—involve uncertainty. It offers a framework to quantify qualitative outcomes and enhance decision-making in contexts like product introduction [42,43]. For products with subjective attributes, such as comfort or quality, the model can capture consumer perceptions before purchase. For example, the value of the comfort of a new shoe can be modeled through elicited subjective preferences. Our model can also adapt to scenarios with unknown probabilities [44]. In these cases, it will provide insight into decision rules by quantifying qualitative aspects and generating more informative payoff matrices.” Lines: 252-260
      iii. “Nevertheless, our results indicate that ambiguity attitudes are consistent across domains when studied simultaneously. Additionally, we provide evidence that experience influences this association. Just as perceived risk shapes risk-taking behavior [17], perceived ambiguity can affect the subjective value of outcomes.” Lines: 274-278
3. Expt 1 Methods: There are a range of exclusions described sequentially throughout the methods section. Please have a clear statement of the initial sample size and final sample size (e.g., 66) in the section on participants. Similarly, while transparency on the full procedures is appreciated, it would be useful to highlight which data (taken from which of the three sessions) was specifically used for this research. For example, am I correct that the data used here was not mixed across behavioral and scanner sessions?
   a. Response: we rearranged the methods to make it more straightforward and reduce ambiguity. You are correct, all decision-making data were collected on the first day.
   b. In the text:
      i. “A sample of sixty-six individuals with valid data was drawn from one hundred and one adults (48 females; age range = 18–89; mean 52.97, SD ±22.41) who were screened for the experiment.” lines 345-347
      ii. “All task data reported here were collected on the first session” lines 360-361
      iii. “In brief, in session 1, participants completed the decision-making task (reported here) and a reversal reinforcement learning task (not reported here).” lines 362-364
      iv. “Valid data from three hundred and thirty-two out of four hundred and four adults (212 females; age range = 20–80; mean 49.362, SD ±14.849) who were recruited using Amazon Mechanical Turk (mTurk) was analyzed.” lines 411-413
4. Expt 2 Methods: Please provide a brief description of points of distinction between E1 and E2. (It feels odd to have to hunt up a preprint to understand the similarity of the methods.)
   a. Response: in the revised manuscript, we add an additional description of the online study.
   b. In the text: “Participants completed tasks similar to those described above, following the same procedures as the in-person sample, with two exceptions [33]. First, each lottery was presented twice instead of four times. Second, an additional condition of 100% ambiguity was introduced..” lines 419-422
5. Modeling results: It seems notable that the Estimated Value model was ranked above the No-subjective Parameters in both the monetary and medical tasks similarly. In some sense, that highlights the importance of modeling the individual difference value curves across an ordinal scale regardless of whether that scale is implicitly or explicitly defined, correct? It seems the paper’s framing could be flipped, in that it implies that even seemingly quantitative decisions are better understood through modeling their qualitative aspects.
   a. Response: Yes, this is indeed one of our key claims. Our findings suggest that even seemingly quantitative decisions, such as monetary ones, benefit from modeling subjective, qualitative aspects. This highlights the importance of capturing individual differences in subjective value curves, regardless of whether the scale is implicitly (qualitative) or explicitly (quantitative) defined. We now include this explicitly in the Discussion.
   b. In the text: “Our results emphasize that decisions traditionally considered quantitative, such as monetary choices, are better understood when accounting for subjective, qualitative aspects of the outcomes. The consistent performance of the Estimated Value model across both monetary and medical tasks suggests that individual value curves, even on ordinal scales, play a critical role in shaping decision-making. This finding supports the idea that qualitative elements underpin decision-making processes across domains, regardless of whether the outcomes are explicitly or implicitly defined.” lines: 297-304
6. Results / Figure 2: The way that the medical and monetary findings are plotted may be misleading. The medical outcomes do have a step scale that’s faithfully reported as fixed intervals on the x-axis in panels A/C. However, it seems odd to have monetary increments of differing amounts plotted in similarly spaced intervals in panel B/D, and this also hides a nice feature of the data, which is an apparent diminishing marginal return as the amount of money increases. A more worrisome interpretation though, is that the linear trend across all four panels could show that participants simply coded both types of stimuli as four levels/steps of outcomes.
   a. Response: Thank you for this - we debated these two options, either to use the x-axis as numeric or transforming it to categories. We have opted to use the categories as we plotted the outcomes from the estimated model, which treated the data as categorical, and thus, we felt we are being more in-line with the data. However, since we agree that there is also merit for a cardinal representation of the x-axis, we now also include a supplementary figure with this view.
   b. In the text: “See supplementary eFigure 4 for an illustration of the curvature in the monetary domain.” Figure 2 ligand
7. Results : Given that participants in Experiment 1 could be roughly split by whether or not they had a history of major surgery, it would be very useful to compare the correlation between the medical and monetary ambiguity coefficients between these groups to better speak to the potential qualitative comparison across domains.
   a. Response: This is an interesting question. While not the focus of this paper, and with the limitation of a reduced number of participants, we now include the additional tests in the supplementary results. In summary, the association between ambiguity attitude in the monetary and medical domains for those who had a history of surgery is much higher. However, there was no robust difference between the groups. Thus, while our data suggest that previous experience may increase the association between the 2 domains, assuming all had experience with money, more data is needed to verify it.
   b. In the text: “Interestingly, the cross-domain association is stronger among individuals with a history of surgery (see supplementary results), suggesting that experience with potential outcomes may partially shape ambiguity attitudes. .” Lines: 199-201
8. Results : Healthwise, there seems to be a natural psychological/categorical break between “needs a wheelchair” and “doesn’t need a wheelchair” – is that visible in the data? In addition, to better understand the behavioral baseline, what was the estimated valuation for “no effect” as per the model estimates or the data itself?
   a. Response: The categories were designed by a physician to reflect a gradual, almost linear increase in improvement levels. We did not model the “no effect” category, as it serves as the reference point, representing no change from the current state, similar to a $0 monetary outcome. Just as receiving $5 reflects a $5 increase from the current wealth state, a slight improvement in the medical domain is evaluated relative to the current medical state. Since “no effect” represents the baseline or reference state, it can be treated as 0, functioning as a constant across both options and not influencing the comparison.
9. Discussion/contribution : Summarizing some of the points made above – the benefit of the subjective modeling is clear, and useful. Overall, the model and data here offer important findings, but may be more limited in what they show than what is claimed in the introduction and discussion. This is primarily because the medical stimuli are not wholly qualitative, but rather coded, described, and presented in monotonic increasing steps. As such, it would be helpful to have a discussion of how much they do or do not advance our understanding of qualitative value over the monetary amounts, which clearly have their own qualitative subjective value. While this may be one step towards more qualitative models, it may not be able to account for less linear or well defined domains, or outcomes with mixed-valence attributes contributing to their values.
   a. Response: Thank you so much for the positive evaluation. In the revised manuscript, we outline and emphasize more that the current model only tests objectively ordered qualitative outcomes. While the model has the ability to estimate values to non-ordered outcomes it still needs to be tested.
   b. In the text:
      i. “To align with the monetary task, these outcomes were created on an ordinal scale.” Lines: 400-401
      ii. “We assumed the outcomes were ordinal (e.g., slight improvement =< moderate improvement), and thus, we modeled the subjective value of each outcome as the sum of the subjective value of the preceding outcomes plus an additive value representing the improvement.” lines: 132-135
      iii. “This study aimed to quantify the values of qualitative outcomes and to characterize individual uncertainty attitudes when making choices between ordinal outcomes.” Lines: 206-208

# Reviewer #2:

1. The authors apply sophisticated computational modeling to decision making under risk and ambiguity, comparing alternative models. There are a number of notable results, among them that qualitative outcomes can be modeled using techniques also applied to quantitative outcomes and that ambiguity attitudes generalize across very different domains (money and medical outcomes). The choice of medical outcomes is especially important (degrees of recovery due to standard treatment, slight improvement, compared to moderate/major/complete recovery). Similar results are obtained across independent samples (online and in-person). The methods are rigorous (e.g., counterbalancing quantitative and qualitative decisions, etc.).
   a. Response: We thank the reviewer for their kind words.
2. The main issue with the current version of the manuscript is that the mechanisms underlying the models are not emphasized sufficiently. The authors do not sufficiently connect the models here to the decision-making literature in terms of the claims and findings in that literature. For example, what are the theoretical assumptions of this version of the classic utility model, how do these models relate to prospect theory, etc. Also, walking the reader through some well-chosen concrete examples would greatly help readers appreciate what it means to support alternative models. The authors should also give the reader a sense of the major progress of ideas in decision theories based on empirical tests. The author should explain how the current modeling results cohere with the larger decision-theory literature.
   a. Response: In the revised manuscript, we try to provide a more comprehensive view of the literature and theories that led to this study. In the discussion, we try to place our results in a broader context. We discuss how our model can explain anchoring, Reference Dependence, and Framing effects.
   b. In the text:
      i. “Current models, such as cumulative prospect theory [22], are not equipped to handle qualitative outcomes.” Lines:51-53
      ii. “Although models like cumulative prospect theory [22] and the Kőszegi-Rabin model [46] account for reference points, they assume stable relationships between outcome values along a continuous curve. These models do not capture how adding new options can shift the perceived value of existing choices, as seen in behavioral phenomena like the decoy effect [47,48]. In the decoy effect, introducing an additional option that is slightly inferior to an existing choice makes that choice more attractive. For example, if participants must choose between $5 for sure or a chance for $25, adding a new $20 option could make the $25 option seem more appealing by comparison. These effects highlight the limitations of classical models, which assume that more data simply refine curve parameters without altering the relationships between outcomes. In contrast, our Estimated Value model can capture these categorical and contextual shifts, providing a more nuanced understanding of how people evaluate options in dynamic decision-making scenarios.” Lines: 283-295
3. In addition, the modeling could be made much more accessible to a broader range of readers. Although this is a computational journal, the potential readership for this work is far broader than those who are highly familiar with this approach to modeling. For example, what do the 225.65 parameters of the estimated value model represent—how are they interpreted (Table 2)?
   a. Response: Thank you for your insightful feedback. We agree that improving accessibility will benefit a broader audience. To clarify, the value 225.65 in Table 2 represents the effective number of parameters (p_loo) for the Estimated Value model, not the literal number of parameters. This value reflects the model’s flexibility—how much it adapts to the data—rather than the raw number of parameters. Importantly, p_loo is an estimate based on the number of data points and the model’s ability to explain patterns without overfitting. The same model applied to a larger dataset (i.e.,the value for the online sample) will have a higher p_loo value, as it would need more flexibility to capture additional variability in the data. Conversely, a smaller dataset would constrain the model, resulting in a lower p_loo.

We have revised the manuscript to make these concepts more accessible to readers less familiar with computational approaches and clarify the relationship between model complexity, data size, and p_loo.

b. In the text:
   i. “Additionally, LOO inherently accounts for model complexity through the effective number of parameters, ensuring that models are not rewarded for overfitting.” Lines: 454-456
   ii. “**Rank:** The position of a model based on its LOO (Leave-One-Out Cross-Validation) score, with 0 indicating the model with the best predictive performance. **LOO**: Expected log pointwise predictive density (ELPD), which measures how well a model can predict unseen data. A higher LOO score reflects better model fit and generalizability. LOO also accounts for model complexity to avoid overfitting—ensuring that more flexible models must demonstrate sufficiently improved predictive accuracy to justify their complexity. **p_loo**: Effective number of parameters - representing the model’s complexity. Higher values indicate that the model has more flexibility in fitting the data. However, this is not a literal parameter count—it reflects the balance between the number of data points and how much the model adapts to capture meaningful patterns. The same model applied to a larger dataset would have a higher p_loo, as more data introduce additional variability to capture. **d_loo**: Difference in LOO between a given model and the best model (ranked 0). This shows how much worse the predictive performance is compared to the top-ranked model. **Weight**: Model weights based on the LOO scores, indicating the relative importance of each model. These weights help in model averaging, with higher weights given to models that show better predictive performance. **SE**: Standard error of the LOO estimate, reflecting the uncertainty in the score. Larger SE values indicate greater variability in the model’s predictive accuracy across different data points.” Legend Table 2

4. Similarly, regarding simulations, it is not clear how the reader should take the message that one model is best (classic utility) but another model (estimated value) showed superior performance as noise increased.
   a. Response: This indeed was not explained clearly. Using simulation, we can control the data generation function and test different parameters. We can use the different models to test parameter recovery and to try and explain the data. With little noise, we assume that a model closer to the data generation function should be better at parameter extraction. Thus, we assume it will show better fit to the data. Once we introduce a large amount of noise in the choice selection, the difference between the data generation function and the actual data generated increases, and the model should show a worse fit to the data. A model that can still find the underlying process is more robust to noise or can accommodate different choice selection mechanisms. We try to clarify it further in the revised manuscript.
   b. In the text:
      i. “The estimated model has more degrees of freedom. Hence, a better fit could result from overfitting [25]. One way to ensure that the results represent real phenomena rather than overfit is by using simulations [26]. We simulated data (Methods) to assess the fit of the Estimated values model in scenarios where it should underperform (low noise) and overperform (high noise). The simulation results demonstrate how noise levels influence model performance. With lower noise (0.1), where the data generation function is closer to the utility curve, the classic utility model provided a better fit. However, as noise increased to 0.3 and 0.5, the estimated value model consistently showed superior performance, regardless of the number of participants. Specifically, at higher noise levels, the estimated value model had higher LOO values and weights across different sample sizes. For detailed results, see supplementary results.” Lines 179-190
      ii. “By controlling the data generation function, we assessed the performance of different models. If the estimated model outperforms the utility function, even when the data were generated by the utility model, it suggests that the estimated model is insufficiently penalized for its complexity, indicating a risk of overfitting. Using the utility function (Equation 1), we simulated datasets with varying parameters, controlling for noise levels and sample sizes. Each selection in the simulation included noise drawn from a normal distribution with a mean of 0 and a standard deviation determined by the simulation. Additionally, each subject had unique risk (α) and ambiguity (β) parameters. To ensure model convergence, we constrained risk attitudes to the range [0.1, 1.6] and ambiguity attitudes to [-1.4, 1.4]. We tested the following sets of parameters (N, noise): (30, 0.1), (30, 0.3), (30, 0.5), (60, 0.3), (60, 0.5), (120, 0.5), (300, 0.5). We then evaluated the utility function and the estimated value model (Equation 4) using LOO scores and weights to compare their ability to fit the simulated data.” Lines: 424-436
      iii. “The simulation results demonstrate how noise levels influence model performance. With lower noise (0.1), where the data generation function is closer to the utility curve, the classic utility model provided a better fit.” Lines: 183-186
      iv. “As expected, in scenarios with low noise levels, where the data generation function closely mimicked the utility curve, the Classical Utility model outperformed the Estimated Value model.” Lines: 306-308
5. Overall, good models should explain as well as “predict” (simulate) out of sample; the best models also make predictions to new situations (ideally counterintuitive predictions that distinguish explanations) that reveal underlying mechanisms. How do the current models map onto the aforementioned desirable features? (What are the “insights into the processes underlying participants’ decision-making?”) What are novel predictions from the supported models? What do the supported models tell us that is new about the brain or the psychology of decision-making? In this connection, being able to model qualitative outcomes is quite an accomplishment, and it seems to map onto recent theory that distinguishes qualitative gist representations from precise quantitative representations—how do the current results speak to those relevant constructs?
   a. Response: Our models aim to provide both explanatory and predictive power. By quantifying qualitative outcomes, we capture underlying decision-making processes that are typically missed by classical models. One key insight is how participants assign subjective value to qualitative attributes, revealing the complexity of decisions beyond numerical comparisons. This aligns with theories distinguishing between qualitative "gist" representations and precise quantitative representations, highlighting that people often rely on subjective impressions, especially under uncertainty. Our model suggests that people treat options as categorical rather than cardinal values, and thus can explain effects such as anchoring and decoy effects. That is, how the introduction of additional categories can change the value of current categories.
   b. In the text: “The field of healthcare, particularly medical decision-making, is a critical area of application, where ongoing debates address the level of information patients receive and how they use it [35]. The fuzzy-trace theory posits that patients often hold only a gist representation of the outcomes they are considering [37]. Our data can be viewed as an attempt to estimate this gist, with our Bayesian approach allowing us to preserve these values as distributions rather than forcing them into point estimates. This method captures a range of possible interpretations, providing a nuanced understanding of patient decision-making. Furthermore, the increased cross-domain association in ambiguity attitudes observed in individuals with prior surgical experience supports the transition from gist to verbatim processing [38]. This finding suggests that personal experience may reinforce a shift towards more detailed, quantitative representations, enriching our understanding of how experience shapes the processing of uncertainty in critical health decisions.” Lines: 239-251
6. The first two paragraphs of the Discussion are very good. The rest is good too but needs to be developed a bit more: “Without the assumptions of specific relationships among them” undersells this strength of the research; the methods here allow the relationships to be discovered rather than assumed.
   a. Response: Thank you so much for this. We completely agree, and in the revised manuscript, we will try to explore this concept a bit more.
   b. In the text: “This approach allows for the use of different categories without the assumptions of a specific distance between them.” Lines: 225-226
7. The authors need to explain more about why risk attitude measures “require equating the outcomes across domains” but ambiguity measures do not, given how similarly they are operationalized.
   a. Response: This is an important point, which we indeed did not explain clearly. Risk attitudes, by definition, rely on outcome value. For example, a participant who prefers a 50% chance of $12 over $5 for sure and also prefers a slight improvement for sure over a 50% chance of moderate improvement. There is no way of comparing this participant’s risk attitude across domains. The participant is willing to take a risk in the monetary domain, not in the medical domain, but may reverse their preference if, for example, the risky monetary outcome was $20. Conversely, ambiguity attitudes are measured as the difference between a choice of an ambiguous option and a choice of a risky option with a 50% chance of obtaining the same outcome. A participant who is willing to accept a 50% chance for $12 but not an ambiguous chance of obtaining the same amount exhibits ambiguity aversion. In other words, ambiguity attitude is defined in a way that does not depend on outcome magnitude.
   b. In the text: “Our focus on ambiguity - rather than risk - attitudes in this study warrants an explanation. Technically, in the Estimated Value model, risk attitudes are not quantified separately but are integrated into the estimated values (Equation 4). More broadly, the measure of **risk attitude** is tied to the outcome value. For example, a participant’s preference for a 50% chance of $12 over a guaranteed $5 reflects how they value different monetary amounts. Similarly, choosing a guaranteed “slight improvement” over a 50% chance of a “moderate improvement” reveals how they value medical outcomes. In both cases, **risk** is inherently linked to the value of the outcome. In contrast, **ambiguity attitudes** are assessed by measuring how participants respond to uncertainty within the same domain, in relation to risk, independent of outcome value. This distinction allows us to compare the **effect of ambiguity** across different domains, such as monetary and medical decisions.” Lines: 261-272
8. What does “follow a specific curve” mean? There are many ways that could be interpreted.
   a. Response: what we tried to say is that the rate of change doesn’t follow an elementary function (e.g., linear, exponential, or logarithmic). In the revised manuscript, we clarify this.
   b. In the text: “While the utility model we used is constrained to follow a specific functional form [45], our estimated model is more flexible and can adapt to the data.” Lines: 281-282
9. Naturally, having more degrees of freedom is a double-edged sword; why is that better here in light of prior findings?
   a. Response: We completely agree, and apologize for not being clear on this. We do not claim having more parameters is better; rather, we penalize our model for being more complex. However, we claim that the fit is robustly better, and thus warrant the use of extra variables.
   b. In the text: “Additionally, LOO inherently accounts for model complexity through the effective number of parameters, ensuring that models are not rewarded for overfitting.” Lines: 454-456
10. Real participants’ data are not necessarily more noisy in the sense of random fluctuation so it is not clear why simply being robust to more “noise” (as opposed to systematic variation) is good.
    a. Response: We agree with this argument. By “noise,” we meant that real participant data are generated using an unknown function. The robustness to noise relates to the simulation where the function that generates the data is known. Once more noise is in the system, the generation function becomes obscure, and our model is better at explaining this data from an obscure data generation function.
11. The authors make a good point that “This result highlights how the LOO algorithm effectively penalizes the Estimated Value model for its extra complexity.” On the one hand, this raises the issue that models should not be penalized for parameters that other work shows must be included to account for specific results. On the other hand, accommodating these parameters could distort results for needed parameters in a specific dataset. The authors should bring such considerations to bear (about penalizing models for assumptions shown to matter in other research) throughout in evaluating the models.
    a. Response: The penalization promotes parsimony and prevents overfitting. Without accounting for the complexity of established models, newer models would need to vastly outperform them to justify any complexity. Penalization ensures fair comparisons, particularly across datasets. Accordingly, we evaluated our models using a replication sample to ensure robustness.
12. The authors make a couple of excellent points about the estimated model allowing for unique curvatures for each set of outcomes. The authors need to give examples of empirical effects that require that assumption and relate this statement to specific prior models (e.g., cumulative prospect theory, Kőszegi-Rabin, etc.). Concrete examples of decision problems and findings help.
    a. Response: This point and point 2 are excellent suggestions. In the revised manuscript, we discuss our model in relation to other models, such as cumulative prospect theory and Kőszegi-Rabin. We discuss the framing effect and the effects that can be explained by our model more.
    b. In the text: “Although models like cumulative prospect theory [22] and the Kőszegi-Rabin model [46] account for reference points, they assume stable relationships between outcome values along a continuous curve. These models do not capture how adding new options can shift the perceived value of existing choices, as seen in behavioral phenomena like the decoy effect [47,48]. In the decoy effect, introducing an additional option that is slightly inferior to an existing choice makes that choice more attractive. For example, if participants must choose between $5 for sure or a chance for $25, adding a new $20 option could make the $25 option seem more appealing by comparison. These effects highlight the limitations of classical models, which assume that more data simply refine curve parameters without altering the relationships between outcomes. In contrast, our Estimated Value model can capture these categorical and contextual shifts, providing a more nuanced understanding of how people evaluate options in dynamic decision-making scenarios.” Lines: 283-295
13. Excellent measurement points are made in the last two paragraphs.
    a. Response: Thank you so much for this.
14. In summary, this is a sophisticated modeling approach to prediction that accommodates different kinds of uncertainty, which provides ways to quantify qualitative outcomes and to compare ambiguity attitudes across monetary and medical outcomes. These are impressive accomplishments, but they will not be accessible to, and sufficiently appreciated by, many readers if not explained in light of the prior literature and mechanisms as discussed above.
    a. Response: We thank the reviewer for their comments and suggestions. We hope that the revised manuscript will be more transparent and more approachable to larger audiences.
